# Evaluation of CTRL: a web application for dynamic consent and engagement with individuals involved in a cardiovascular genetic disorders cohort

**DOI:** 10.1038/s41431-023-01454-1

**Published:** 2023-09-14

**Authors:** Matilda A. Haas, Evanthia O. Madelli, Rosie Brown, Megan Prictor, Tiffany Boughtwood

**Affiliations:** 1 Australian Genomics, Parkville, VIC 3052 Australia; 2 Murdoch Children’s Research Institute, Parkville, VIC 3052 Australia; 3 Melbourne Law School, University of Melbourne, Parkville, VIC 3010 Australia

**Keywords:** Outcomes research, Genetics research

## Abstract

There has been keen interest in whether dynamic consent should be used in health research but few real-world studies have evaluated its use. Australian Genomics piloted and evaluated CTRL (‘control’), a digital consent tool incorporating granular, dynamic decision-making and communication for genomic research. Individuals from a Cardiovascular Genetic Disorders Flagship were invited in person (prospective cohort) or by email (retrospective cohort) to register for CTRL after initial study recruitment. Demographics, consent choices, experience surveys and website analytics were analysed using descriptive statistics. Ninety-one individuals registered to CTRL (15.5% of the prospective cohort and 11.8% of the retrospective cohort). Significantly more males than females registered when invited retrospectively, but there was no difference in age, gender, or education level between those who did and did not use CTRL. Variation in individual consent choices about secondary data use and return of results supports the desirability of providing granular consent options. Robust conclusions were not drawn from satisfaction, trust, decision regret and knowledge outcome measures: differences between CTRL and non-CTRL cohorts did not emerge. Analytics indicate CTRL is acceptable, although underutilised. This is one of the first studies evaluating uptake and decision making using online consent tools and will inform refinement of future designs.

## Introduction

Since first proposed more than a decade ago, dynamic consent continues to be debated while implementations have been trialled [1, 2]. Dynamic consent has been proposed as a consent and communication platform for various use cases including large-scale, longitudinal research, genomics and biobanking. Its purported benefits have included enhanced decisional autonomy and control, recruitment and retention of participants, trust and ongoing engagement with research [3], with the ability to meet increasingly stringent regulatory requirements [4]. Dynamic consent also reflects a broader move from paper-based to electronic forms of consent for pragmatic reasons including better record keeping and increasing use of personal technology [5] and achieving greater geographical representation of individuals in research [6]. Some have challenged the claims made of dynamic consent, including questioning the role of individuals using dynamic consent in making governance decisions and whether it increases burden or contributes to ‘consent fatigue’ [7, 8]. With others, we have previously called for structured formal evaluation and reporting of the dynamic consent model, to resolve competing claims as to its effects, and to build the evidence base to inform its use and refine the approach [3].

We previously reported on the digital consent and engagement platform inspired by dynamic consent, a web application called CTRL (‘control’) [9]. We now report on a pilot implementation and evaluation of CTRL in the Australian Genomics Cardiovascular Genetic Disorders cohort study [10]. Previous studies have sought participants’ views about interacting with dynamic consent tools, concluding that participants see value in the ability to change consent preferences [11], but especially value the ongoing engagement it can facilitate [12]. The current evaluation study looks at the characteristics of individuals who independently take up dynamic consent and how they use it, individual choices about return of incidental results (those with potential medical significance or value but beyond the scope of the original test or research question and not intentionally sought), and future research use of their genomic, health and self-reported experience data. It reports on key outcome measures identified in the dynamic consent evaluation framework, consistent with the broader evaluative literature on novel informed consent methodologies in health research settings [3].

## Materials and methods

### Study population

The Cardiovascular Genetic Disorders Flagship (the Flagship) used whole genome sequencing to seek a genetic diagnosis for the cardiac condition of 600 individuals. The Flagship recruited patients and their families with either inherited cardiomyopathies, primary arrhythmia diseases or congenital heart diseases. The study was offered to both adult and paediatric individuals, and recruitment and consent was usually done through a face to face or telehealth appointment with a genetic counsellor, study coordinator or cardiac specialist. Flagship recruitment commenced in April 2019.

### CTRL prospective cohort

Individuals recruited to the Flagship at 12 study sites across Australia were invited to participate in CTRL within a 22-month period (March 2020–December 2021). Each site had its own team involved with recruitment. Following an initial in person, phone or telehealth discussion in which individuals agreed to receive further information about CTRL, an e-mail was sent to them via the study database, REDCap [13, 14], inviting them to register to CTRL online. The invitation email included the individual’s unique study ID and the URL to CTRL, where individuals were able to: update their personal and contact details; make and change consent choices; contact the researchers through a messaging system; view news and information; and track their progress through the study.

A CTRL flyer (Supplementary information) was incorporated into the paper consent form and the e-consent platform used by individuals to enrol in the study, aiming to increase awareness of CTRL even if they were not informed about it by their study recruiter.

### CTRL retrospective cohort

After the recruitment period into the Flagship ended in December 2021, eligible currently enroled individuals were (re)contacted via e-mail to invite them to register to CTRL. Eligible individuals from 16 recruitment sites included individuals who: 1) had not already registered to CTRL; 2) had not been withdrawn; 3) had not declined CTRL participation previously; and 4) whose genetic counsellor deemed contact to be appropriate.

Individuals were invited to register to CTRL by a REDCap e-mail invitation. Individuals who had been offered CTRL in person previously and agreed to participate but did not register were sent one e-mail invitation. Individuals who had not been offered CTRL by a study recruiter previously were sent an initial invitation and one reminder after one month. The CTRL flyer was attached to the e-mail for their reference.

The prospective and retrospective recruitment strategies are outlined in Supplementary Figure 1. Careful not to overburden participants with contact, so far, we have only contacted participants in relation to their CTRL registration if 1) they registered to CTRL but did not make any consent selections, and 2) if they requested notification when their data was shared for secondary research. They could initiate contact with us at any time through CTRL.

### Preferences for return of results and future sample and data use

As part of registration to CTRL users select optional responses (‘Yes’, ‘No’, ‘Not Sure’) to consent questions about their preferences for return of results, as well as secondary use data sharing [9] (and Supplementary information). There were 17 items across two optional consent sections.

### Outcome measures relevant to CTRL

Individuals enroled in the Flagship were asked to complete surveys online via a REDCap link sent to their email address at baseline, and 1, 6, 12, and 24 months post-genomic test result delivery. To compare the experiences of the cohort who used CTRL with the cohort who did not (‘non-CTRL’), validated survey questions on satisfaction with the study [15], decisional regret [16], trust in research(ers) [17] and understanding of genomic testing [18] were included in the surveys, which also asked demographic questions (Supplementary information). These outcome measures were selected considering outcome domains commonly used in research evaluating informed consent [19, 20], and although they pre-dated the publication of the ELICIT study findings which specified a core outcome set for such research, in practice they were well aligned with its recommendations [21]. Pragmatism was also a factor in the selection of outcome measurement tools; those chosen were relatively few and deliberately brief.

### Data analysis

Chi-square goodness for fit tests were performed in StataIC 17.0 [22] to compare characteristics of individuals who did or did not register to CTRL, either prospectively or retrospectively. The expected values were adjusted based on the number of individuals invited to register to CTRL. To examine if individuals of a certain age group are more likely to register to CTRL, individuals were grouped into 0–25, 26–50, and over 50 years. Education levels were grouped into ‘Higher education’ (post-graduate degree, bachelor’s degree, TAFE/College also known as vocational training/trade certificate) and ‘No higher education’ (year 12 HSC, still studying, did not finish high school). Chi-square was also used to determine if obtaining a genetic diagnosis resulted in more participants signing up to CTRL after receiving an invitation retrospectively. Expected values were adjusted based on the diagnostic rate of the overall Flagship. StataIC 17.0 was used to analyse responses and report descriptive statistics of surveys and consent preferences. Due to the limited number of responses from parents/guardians who completed the surveys on behalf of paediatric individuals, parent/guardian responses were excluded.

### Website analytics

Google analytics was used to understand user engagement with CTRL. CTRL users were linked to Google analytics based on registration time. Metrics to investigate use of the website included device used, session duration, pageviews per session, number of sessions and time spent on pages. Data were extracted from Google analytics and descriptive statistics were reported using Microsoft Excel version 16. Data from the prospective and retrospective cohorts were analysed separately.

## Results

### CTRL prospective cohort acceptance rates

**Fig. 1:**
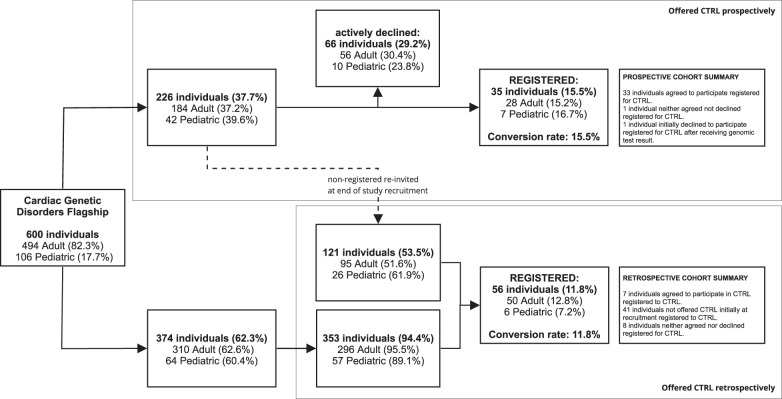
Recruitment design and acceptance rates. The prospective cohort was ascertained via the top pathway, the retrospective cohort via the bottom pathway. Individuals who originally accepted the in person offer to register to CTRL but did not sign up were sent an e-mail invitation at the completion of the recruitment period, and those registrations were included in the retrospective cohort (represented by dashed line). The rest of the retrospectively invited group is 353 individuals who were recruited into the Flagship but were never offered CTRL. The percentages of the prospective and retrospective registered groups were calculated based on the individuals who were invited to participate to CTRL. Other percentages are based on the previous step in the figure.

For the prospective cohort, 226 were offered the opportunity to sign up to CTRL at their recruitment appointment, of whom 66 (29.2%) declined and 37 (16.4%) individuals created a registration (Fig. 1). Two individuals (0.3%) subsequently withdrew from the Flagship, so their CTRL registrations were deleted. This resulted in a final number of 35 (15.5%) individuals who prospectively registered to CTRL after learning about CTRL during recruitment and having received one email outlining the purpose of CTRL and how to register. There were 121 (53.5%) individuals who did not sign up but were eligible to be recontacted after completion of study recruitment as they did not actively decline.

### CTRL retrospective cohort acceptance rates

The 121 individuals that did not respond to prospective recruitment were emailed information about how to register once more, within a period of 8–24 months after their first invitation. From this, another 7 (5.8%) individuals registered.

Three hundred and seventy-four individuals were not initially offered the opportunity to register to CTRL at recruitment, either because CTRL was not yet available, or because of other operational reasons at the recruitment site. It was appropriate to ask later because they still had the opportunity to exercise choices about return of results and/or secondary data use. Of these, 353 (94.4%) were emailed with the details they needed to register at the completion of study recruitment. Twenty-one individuals (5.6%) were not contacted because they did not meet eligibility criteria. Thirty-five (9.9%) registered after the first email. Those who did not register received one follow up email four weeks after the first, which led to another 14 (4%) registrations. In total 56 (11.8%) individuals signed up retrospectively.

### Characteristics of the cohort

The total sample size of the study is 91 individuals (35 in the prospective cohort and 56 in the retrospective cohort) who registered to CTRL. The CTRL cohort consisted of 78 adults and 13 parent/guardians of paediatric individuals (14.3%), compared to the overall Flagship cohort which was 17.7% paediatric individuals. Of those offered CTRL, 56 (30.4%) adults actively declined, and 10 (23.8%) parent/guardians of paediatric individuals actively declined. The median age of individuals in the prospective cohort was 43 years while the retrospective cohort median age was 42 years, compared to the non-CTRL cohort age of 40 years. The median age of active decliners was 38 years (Table 1). The proportions of individuals who registered to CTRL prospectively or retrospectively did not differ by age (*p* = 0.5591, *p* = 0.9461).

**Table 1:** Cohort characteristics.

Characteristic	CTRL (prospective)	CTRL (retrospective)	Non-CTRL	Active decliners
Age in years at consent median (range)	43 (0–69)	42 (0–72)	40 (0–82)	38 (0–72)
0–25	9	14	115	17
26–50	15	27	206	36
>51	11	15	123	13
Gender number (%)				
Male	17 (48.6)	39* (69.6)	238 (53.6)	32 (48.5)
Female	18 (51.4)	17 (30.4)	206 (46.4)	34 (51.5)
Non-binary	0	0	0	0
Education level attained number (%)				
Post-graduate degree	5 (15.6)	14 (29.8)	40 (13.9)	8 (18.6)
Bachelor’s degree	10 (31.3)	12 (25.5)	69 (24.0)	14 (32.6)
Trade/vocational training	9 (28.1)	11 (23.4)	91 (31.7)	9 (20.9)
High school certificate	3 (9.4)	5 (10.6)	37 (12.9)	7 (16.3)
Still studying	1 (3.1)	1 (2.1)	9 (3.1)	1 (2.3)
Did not finish high school	4 (12.5)	4 (8.5)	41 (14.3)	4 (9.3)
Genetic diagnosis received number (%)				
Prior to CTRL registration	0	24 (42.9)		
After CTRL registration	11 (31.4)	0		

Characteristics of individuals in the prospective, retrospective, non-CTRL and active decliner groups. The median age at the time of consent and age ranges, the gender (a non-binary option was not selected by any individuals), the level of education reported by individuals involved in the study, the proportion of individuals in the retrospective cohort who had received a diagnostic or non-diagnostic result at the time of registration to CTRL compared are reported. One individual who initially actively declined the invitation to CTRL went on to create a registration at the time they received their genomic test result. This individual is included in the prospective and active decliner columns. (*p < 0.05).

Eighteen (51.4%) of the prospective cohort identified as female, 17 (48.6%) as male (*p* = 0.7602). Seventeen (30.4%) of the retrospective cohort identified as female, 39 (69.6%) as male, which was a significant difference (*p* = 0.0276). For active decliners, 34 (51.5%) identified as female and 32 (48.5%) as male (Table 1). The most frequently reported education level for the prospective cohort was bachelor’s degree (10, 31.3%), while it was postgraduate degree for the retrospective cohort (14, 29.8%), trade certificate/vocational training for the non-CTRL cohort (91, 31.7%) and bachelor’s degree for the active decliners (14, 32.6%) (Table 1). Education level was not different within groups who registered to CTRL, either prospectively (*p* = 0.5371) or retrospectively (*p* = 0.3046). Education level did not differ between those who registered to CTRL and those who did not (*p* = 0.190). For the prospective cohort (who registered to CTRL near the beginning of the study) no one had received their genomic test results. One individual who initially declined to participate in CTRL subsequently created a registration two days after receiving their test result 11 months later. For the retrospective cohort 24 individuals (42.9%) had been given a diagnostic (pathogenic or likely pathogenic) result prior to their registration, which was not different to those who had not (*p* = 0.1618).

### Individuals’ preferences for future (secondary) research use of health and genomic data

**Fig. 2:**
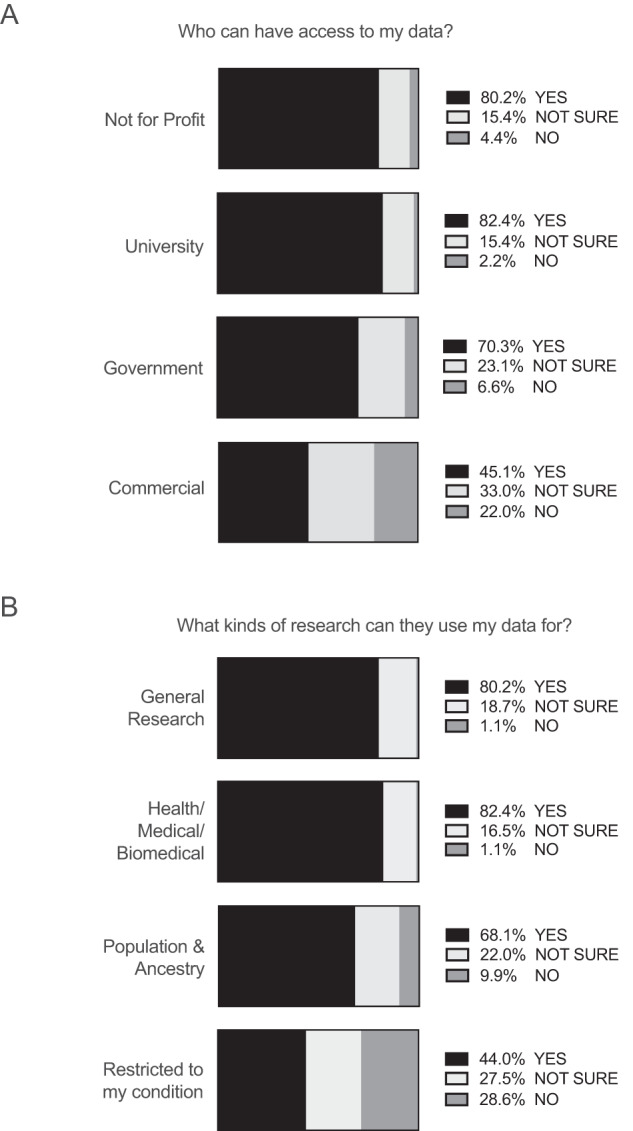
Preferences for future (secondary) research use of samples and data. Answers to questions in consent step 5 (permissions for different types of future research use of samples and data) are reported as percentages (%). Paediatric, adult, prospective and retrospective groups were combined so that the overall cohort choices are shown. **A** Organisations permitted to use data, **B** Types of research data can be used for. The individual group choices, consent step questions and Stata analysis tables are also available to be reviewed in Supplementary information.

Individuals provided answers to questions about permissions for future (secondary) use of their biological samples and study information for other ethically approved research projects (where information includes demographic, health, genomic and self-reported data). The questions (Supplementary information) align with the Global Alliance for Genomics and Health’s Data Use Ontology standard [23]. Most individuals permitted use of their samples and information by not-for-profit organisations (73, 80.2%) and universities (75, 82.4%), while permissions for use by government (64, 70.3%) and the commercial sector (41, 45.1%) were lower (Fig. 2A).

Most individuals were willing to share for general research use and clinical care (73, 80.2%) and health/medical and biomedical research (75, 82.4%) with fewer agreeing to share for population and ancestry research (62, 68.1%). Research was limited to the persons specific condition by 40 individuals (44%) (Fig. 2B).

Overall, 58 (63.7%) people indicated that they wanted to be notified every time their information was shared for new research, but 6 (85.7%) of the prospective paediatric cohort wanted this information. Of the overall cohort, 72 (79.1%) gave permission to share their contact details with other research programs for which they may be eligible (Supplementary Table 1). There were no differences between prospective and retrospective cohorts in the choices they made.

Australian Genomics is sharing its genomic and health dataset with ethically approved projects, following data access committee review. Since this process began, 50 (of the 58) individuals who chose in CTRL to be notified any time their data were shared have been notified of this by email (data not shown). Of those, five individuals have been notified twice. This has not prompted any individuals to log back in (either to make changes to the types of research and organisations they give permission for, or to opt out of receiving such alerts when their data are shared).

### Individuals’ preferences for return of results

**Fig. 3:**
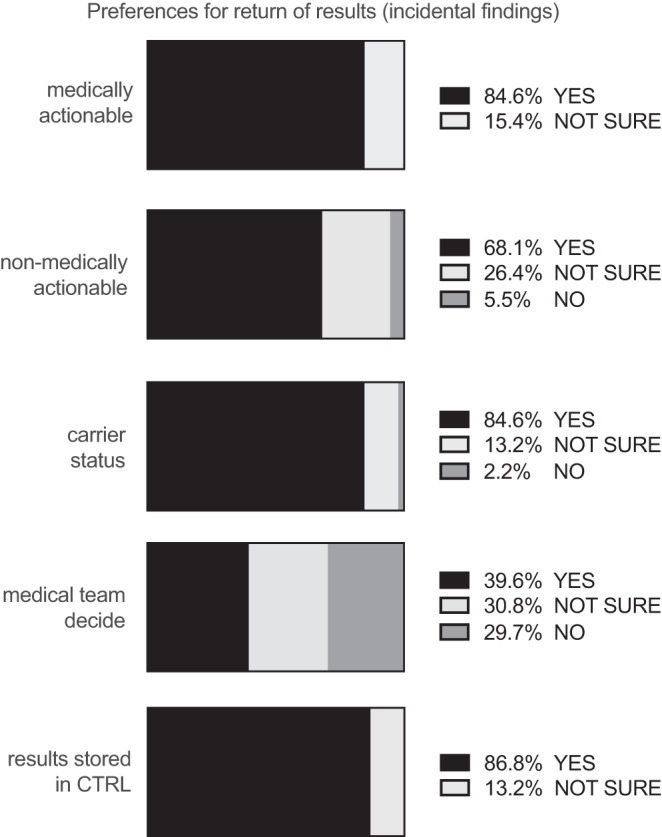
Preferences for return of results. Answers to questions in consent step 4 (preferences for the return of different types of incidental findings) are reported as percentages (%). Paediatric, adult, prospective and retrospective groups were combined so that the overall cohort choices are shown. The individual group choices, consent step questions and Stata analysis tables are also available to be reviewed in Supplementary information.

Individuals provided answers to questions about the return of incidental findings. Most people wanted to know about medically actionable (77, 84.6%) non-medically actionable (62, 68.1%) and carrier status (77, 84.6%) results. Sixty-nine percent of individuals across the cohort wanted all types of incidental results returned to them and 79 (86.8%) wanted a summary of their genomic testing results stored securely in CTRL, if available (Fig. 3).

### Outcome data of CTRL and non-CTRL users

Satisfaction with research experience, decision regret, trust in research(ers) and knowledge of genomic testing outcome measures were not significantly different between CTRL and non-CTRL users at baseline or any subsequent timepoints (Supplementary Figure 2). However, the sample is too small to draw reliable conclusions based on these preliminary data.

### Individuals’ use of CTRL

**Fig. 4:**
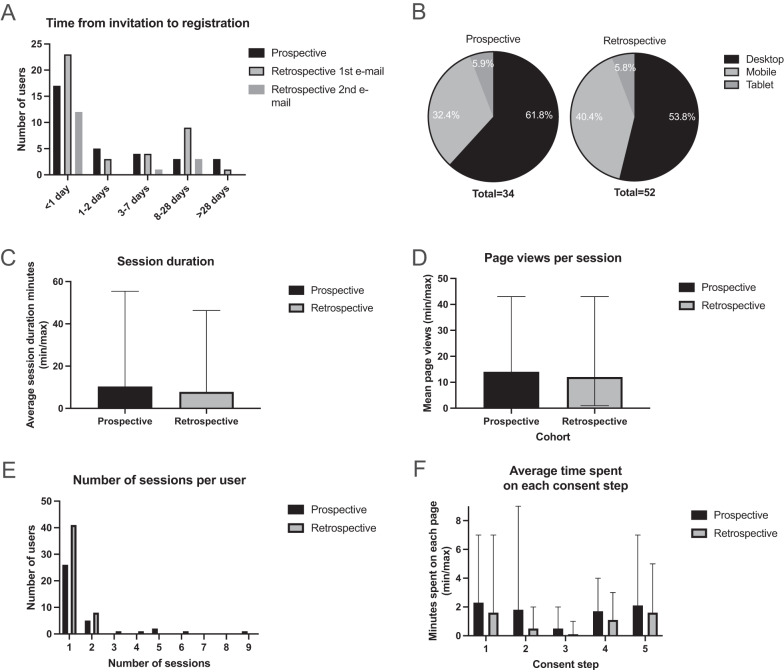
CTRL usage characteristics. **A** The time between invitation to registration for the prospective cohort and the retrospective cohort. For the retrospective cohort, those who registered after the first email or second email are shown. **B** Percentage of individuals in the prospective and retrospective cohort who used different internet enabled devices to register to CTRL. **C** Average first session duration, showing the range (min/max) **D** Average pageviews per session, showing the range (min/max). **E** Number of sessions per user. **F** Average time spent on each consent step. Most individuals in the retrospective cohort accessed a version of CTRL that did not have steps 2 and 3. Questions in each consent step are shown in Supplementary information.

Of the 91 individuals in the overall CTRL cohort, website analytics were unable to be linked for five individuals (one from the prospective cohort and four from the retrospective cohort). Data for the two withdrawn individuals were deleted. For the prospective cohort, 17 individuals signed up within one day of invitation (48.6%), while for the retrospective cohort 23 individuals who signed up did so within one day of the first email (41.1%), and a further 12 individuals (21.4%) within one day of the reminder email (Fig. 4A). Individuals accessed CTRL using a desktop computer (21, 61.8% prospective; 28, 53.8% retrospective), mobile phone (11, 32.4% prospective; 21, 40.4% retrospective), or tablet (2, 5.9% prospective; 3, 5.8% retrospective) (Fig. 4B). The average session duration was 10:22 min for the prospective cohort (range <1–55 min) and 7:51 min for the retrospective cohort (range <1–46 min) (Fig. 4C), (there is a timed logout after 10 min of inactivity). There was an average of 14 and 12 pageviews per session for prospective and retrospective cohorts, respectively (Fig. 4D). Most individuals in the retrospective group were diverted to a version of CTRL with two fewer pages than those who registered prospectively. Across both cohorts, most (67, 78%) individuals logged in for one session. The maximum number of sessions for a user was nine for the prospective cohort and six for the retrospective cohort (Fig. 4E). Most individuals entered their consent preferences during their first session. Over the duration of the study, nine new registrants left all answers on the default option ‘Not Sure’ and were contacted by the study team. Throughout the data collection period (March 2020 to July 2022), no individuals re-entered CTRL and made changes to their initial selections (data not shown).

For the 5-step consent part (Supplementary information), the average time spent on Step 1, which includes a 2:57 minute introductory information video, was 2:30 min for the prospective cohort and 1:57 min for the retrospective cohort (Fig. 4F). This indicated that most users did not watch the video to the end. Aside from the 5-step consent, other pages were not frequently accessed. The number of users who entered additional personal details (that were not automatically transferred from the registration page) was 20 (57.0%) and 34 (60.7%) respectively. Zero users entered the ‘Contact us’ page, and one user in the prospective cohort viewed the ‘News and information’ page (data not shown).

## Discussion

This study provides among the first evaluative data internationally on the implementation of a dynamic consent platform. Of additional note, the cohort is representative of real-world users who enroled in the dynamic consent platform as part of a research study where participation involved undergoing diagnostic genomic testing for a cardiovascular condition. Thus, the individuals in this study were experiencing a significant health issue, as compared to other studies that have tested hypothetical consent interventions, or those that have surveyed public opinion. In this study, 15% of the eligible cohort registered to CTRL. Demographics including age, gender and education level were not different between individuals who chose to register to use CTRL or not. However, significantly more males than females enroled when invited retrospectively. Individuals in both prospective and retrospective cohorts were generally permissive of their information being shared for secondary research, particularly to public organisations involved in research categorised as clinical care, health, medical and biomedical research. Analytics indicated that CTRL was acceptable to users, who were able to navigate it to complete consent choices. Although there were insufficient data to draw robust conclusions, neither research experience (trust, decision regret and satisfaction), nor level of genomics knowledge, were better or worse between the cohort who registered for CTRL compared with those who did not.

The number of individuals in the Flagship who registered to CTRL was 91/600 (15%). This registration rate was anticipated and likely due to several factors: CTRL was introduced as a secondary consent method rather than an alternative primary intervention; sign up to CTRL was completely voluntary and most individuals passively declined, and there were likely differences in whether and how study recruiters approached the CTRL conversation with individuals. Lack of motivation due to experiencing a health issue may have been another factor. Although we did not specifically evaluate reasons why individuals did not sign up in this study, sign up rates in some other dynamic consent implementations have also been low (e.g. 23 participants in 2.5 years for the RUDY Japan study, though acknowledging this project has focused on patient involvement in design so far) [24], in contrast to other longitudinal, registry-based dynamic consent platforms [25, 26].

This study does not support the assumption that the ‘digital divide’ will exclude some groups from using dynamic consent - at least when compared to the rest of the Flagship cohort - as there were no differences in education level, age or gender between groups who used CTRL and those who did not. Ethnicity was not analysed (see below, limitations section). For the retrospective cohort, whether a person had received a genetic diagnosis before the offer of CTRL registration did not appear to influence their decision to sign up to CTRL. We had previously hypothesised that people who had no diagnosis identified and are still seeking answers about their health condition might be more motivated to use a dynamic consent platform to manage access to further research opportunities [27].

It is unclear why significantly more males than females registered to CTRL in the retrospective group. However, males are likely to engage in online activity when seeking information about their health condition [28] and can be motivated by consideration for their health and wellbeing when offered opportunities to self-monitor their condition or study progress [29]. Cardiac patients are often experienced with ongoing self-monitoring and drug adherence programs [30] so may be better adept at interacting with an online tool and allude to altruistic motives for engaging in research [31].

Individuals’ choices about which organisations could access their information for secondary research and the types of research they permitted were not entirely consistent with previous studies. In our study, organisations involved in health and medical research were more permitted than government or commercial organisations, but compared to other studies, individuals in our study were more permissive of secondary research by commercial organisations [32, 33]. The permissiveness of health data sharing in this study could be attributed to differences in perspectives of those with familiarity or personal experience with genetics, as compared to public [34–36]. Our data also show that individual choices about secondary data sharing do differ, which warrants incorporation of more granular decision-making in genomic research.

Our study adds to growing evidence that people only rarely make changes to their choices in dynamic consent platforms [24, 25]. This raises an important question about how much investment should go into development of such platforms if they are underutilised. Our study also suggests dynamic consent may only be the preferred consent mechanism for a minority of participants in research. It is our view that the needs of all groups should be met in research and so consent could be designed so that broad, tiered and dynamic consent options can be offered. E-consent and dynamic consent platforms will continue to increase in the digital age, and they could have many benefits, such as diversifying recruitment in biobanks and population level studies and meeting the needs of First Nations peoples in research participation [36]. More research needs to be done to determine the effect of dynamic consent on participant experience outcome measures, as we were unable to show differences between CTRL and non-CTRL users. This is consistent with the initial evaluation of an online decision aid [37].

We consider ‘consent fatigue’ to include the burden arising from ongoing contact as well as ongoing requests for decision-making. Consent fatigue was not observed in this evaluation. Study data supporting this includes that >90% registered participants completed all consent steps/questions, and no one dropped out without viewing all consent pages. No one contacted us to tell us that they want to receive less contact, and following the receipt of data sharing alerts, no participants have logged back into CTRL to change their preferences so that they do not receive further data sharing notifications.

We did not identify any barriers to using the web app and user acceptance testing had already been undertaken during CTRL development [9]. Individuals who registered to CTRL usually only logged in for one session and worked through the consent steps providing answers to questions. Few people (8%) exited their first session without recording consent choices. Also, no issues were reported, either by selecting request for contact at the bottom of one of the consent steps, via the ‘Contact us’ form, or direct contact with study personnel. While previous studies have reported the importance of dynamic consent portals as a means for contacting the researchers [6], the underutilisation of the CTRL ‘Contact us’ form may reflect the ongoing relationship between the individual and study recruiters in this study. Training and support from the CTRL team were provided to personnel at recruitment sites, but differences between recruitment rates at different sites were demonstrated.

### Limitations

There were several limitations to this study, including low recruitment rates and recruitment only of potentially highly engaged, educated participants who were experienced with health research. There was no randomisation method to decide which individuals to invite to sign up to CTRL, so this was mainly at the discretion of the study recruiter and may have introduced bias. We were unable to include ethnicity/ancestry information as it was not reliably recorded. Finally, the completion rates of the research experience questionnaires were low, so it was not possible to use inferential statistical methods to compare CTRL and non-CTRL groups. Despite these limitations, this study offers novel insights into the characteristics of use of dynamic consent platforms.

### Future directions

This study could be built upon by inviting CTRL users and decliners to participate in further qualitative research. This would inform future refinements and priorities for CTRL. The CTRL codebase is freely available in GitHub. It has been made available to support expansion and application to different use cases. Further studies aimed toward gaining an understanding of different stakeholder perceptions, enablers and barriers, and cost effectiveness are much needed. The potential benefits to researchers are attractive: they include more standardised records of consent, reduced loss of contact with participants (for new or extended consent), clearer governance and interoperability of datasets and compatibility with data sharing processes [9]. However, more research needs to be done to maximise and identify optimal designs of dynamic consent platforms.

## Conclusions

This study provides among the first evaluative data for a dynamic consent platform. We found optional return of results and secondary research use of data choices demonstrate that people do differ in their choices about their participation in research, which warrants providing options for granular decision making to individuals participating in research, with alternatives available to those who want it. Although data were insufficient to draw robust conclusions, there was no indication that individuals who used CTRL were different from non-CTRL users on measures of satisfaction, trust, decisional regret, and knowledge. CTRL will remain active to ensure that individuals’ consent choices are honoured as we share genomic data over time. There may be an opportunity to provide longitudinal results for this study.

### Supplementary information

Supplementary information

## Data Availability

Data from this study is available via contacting Australian Genomics to submit a data access request.

## Code availability

The CTRL code is publicly available at https://github.com/Australian-Genomics/CTRL.
