# Supplementary material for: Evaluation of CTRL: a web application for dynamic consent and engagement with individuals involved in a cardiovascular genetic disorders cohort
Source: Eur J Hum Genet. 2023 Sep 14;32(1):61–8. doi: 10.1038/s41431-023-01454-1 (PMC10772119; doi:10.1038/s41431-023-01454-1)
Supplement: Supplementary file 1 — Supplementary information [file 41431_2023_1454_MOESM1_ESM.pdf]

Supplementary information for:

Evaluation of CTRL: a web application for consent and engagement with individuals involved in a cardiovascular genetic disorders cohort

Matilda A. Haas, Evanthia O. Madelli, Rosie Brown, Megan Prictor and Tiffany Boughtwood

Supplementary Table 1. Preferences for future (secondary) research use of samples and data and return of results. Answers to questions in consent step 4 (preferences for the return of different types of incidental findings) and consent step 5 (permissions for different types of future research use of samples and data) are reported as number (n) and percentage (%). Pediatric, adult, prospective and retrospective groups, as well as the overall cohort choices are shown. The consent step questions and Stata analysis tables are also available to be reviewed in Supplementary information.

Supplementary Figure 1. CTRL as part of the recruitment strategy. Process of recruiting participants to register to CTRL. The top part of the figure refers to the prospective pathway and the bottom part to the retrospective pathway. In both pathways the CTRL flyer and the study database, REDCap, were used but the timing of invitation differed.

Supplementary Figure 2. Research experience of CTRL and non-CTRL users. A) Satisfaction with the study at baseline was 9.03 for the CTRL group (range 3-10, SD = 1.66) and 8.22 for the non-CTRL group (range 2-10, SD = 1.86) out of 10, respectively, and scores remained above 8 in follow up surveys for both cohorts. B) Decisional regret question high score indicates low decision regret (6-30 points). The mean score at baseline was 27.1 (SD = 3.1) for the CTRL group and 26.6 (SD = 3.0) for the non-CTRL group. Scores remained above 22.3 in follow up surveys for both groups. C) Trust in research(ers) questions resulted in a score between 4 and 20 points. The mean score at baseline was 15.2 (SD = 3.1) for the

CTRL group and 15.9 (SD = 3.2) for the non-CTRL group. D) Understanding of genomics questions were scored by the correct number of responses. For both the CTRL and non-CTRL groups at baseline was 8 (SD = 1 and SD = 2, respectively). All graphs report mean and standard deviation (SD), comparing CTRL (adult responses, prospective and retrospective combined) and non-CTRL groups.

Page 4. **CTRL flyer.** The flyer was included in paper-based consent forms used by study recruitment sites, and as an attachment to CTRL invitation emails sent to the retrospectively invited individuals.

Page 5. **CTRL consent step questions as they appear in the version of CTRL used by individuals in this study.**

Page 9. Stata analysis tables for data presented in Table 1, Figures 2 and 3, Supplementary Table 1 and Supplementary Figure 2.

Page 38. **Self-reported experience evaluation measures.** An additional question was included to capture the outcome of valuing the ability to change one's mind. The wording of the Trust scale was also amended, replacing ‘patients’ with ‘participants’. Satisfaction was measured using an 11-point rating scale (0-10). Decision regret and trust in research(ers) were measured using a 5-point Likert-type scale which consisted of 6 and 4 items respectively. For decision regret, consistency was assessed by reversing the scoring of two of the six items that were negatively phrased. Higher score (6-30 points) indicated more confidence in decision (i.e. less decision regret). For the trust in research(ers) questions, the scoring of one of the four items was reversed. Higher score (4-20 points) indicated more trust. The understanding of genomic testing was measured using 10 items with the option to select ‘True’, ‘False’ or ‘Unsure’. The number of correct responses was used to obtain a total score with a range between 0-10.

Supplementary Table 1. Preferences for future research use of samples and data and return of results

Preferences for future research use of biological samples and data

			Not-for-profit research organisations (eg Murdoch Children's Research Institute)	Universities and research institutes (eg The University of Queensland)	Government (eg Australian Government Department of Health)	Commercial companies (eg pharmaceutical companies)	General research use and clinical care	Health/medical/ biomedical research	Research must be specifically related to my condition	Population and ancestry research	I agree to my general health information (eg just my MRIs, blood test or other results) being shared with other research studies that don't need my genomic information	I agree to my self-reported information (eg questionnaire responses) being shared with other research studies that don't need my genomic information	I want to be contacted every time my de-identified DNA sample, genomic, health or self-reported information is shared	I agree to Australian Genomics sharing my contact details with other research projects and clinical trials doing studies I am eligible for
**Prospective**	**Paediatric n (%)**	Yes	5 (71.4)	5 (71.4)	4 (57.1)	3 (42.9)	4 (57.1)	5 (71.4)	4 (57.1)	3 (42.9)	4 (57.1)	4 (57.1)	6 (85.7)	7 (100)
		Not sure	1 (14.3)	1 (14.3)	2 (28.6)	2 (28.6)	2 (28.6)	1 (14.3)	2 (28.6)	3 (42.9)	2 (28.6)	2 (28.6)	1 (14.3)	0
		No	1 (14.3)	1 (14.3)	1 (14.3)	2 (28.6)	1 (14.3)	1 (14.3)	1 (14.3)	1 (14.3)	1 (14.3)	1 (14.3)	0	0
	**Adult n (%)**	Yes	23 (82.1)	24 (85.7)	20 (71.4)	12 (42.9)	24 (85.7)	24 (85.7)	13 (46.4)	18 (64.3)	17 (60.7)	16 (57.1)	18 (64.3)	23 (82.1)
		Not sure	3 (10.7)	3 (10.7)	5 (17.9)	6 (21.4)	4 (14.3)	4 (14.3)	6 (21.4)	5 (17.9)	8 (28.6)	9 (32.1)	5 (17.9)	0
		No	2 (7.1)	1 (3.6)	3 (10.7)	10 (35.7)	0	0	9 (32.1)	5 (17.9)	3 (10.7)	3 (10.7)	5 (17.9)	5 (17.9)
**Retrospective**	**Paediatric n (%)**	Yes	5 (83.3)	5 (83.3)	4 (66.7)	2 (33.3)	5 (83.3)	5 (83.3)	3 (50.0)	5 (83.3)	3 (50.0)	3 (50.0)	4 (66.7)	5 (83.3)
		Not sure	1 (16.7)	1 (16.7)	2 (33.3)	3 (50.0)	1 (16.7)	1 (16.7)	2 (33.3)	1 (16.7)	3 (50.0)	3 (50.0)	2 (33.3)	0
		No	0	0	0	1 (16.7)	0	0	1 (16.7)	0	0	0	0	1 (16.7)
	**Adult n (%)**	Yes	40 (80.0)	41 (82.0)	36 (72.0)	24 (48.0)	40 (80.0)	41 (82.0)	20 (40.0)	36 (72.0)	30 (60.0)	30 (60.0)	30 (60.0)	37 (74.0)
		Not sure	9 (18.0)	9 (18.0)	12 (24.0)	19 (38.0)	10 (20.0)	9 (18.0)	15 (30.0)	11 (22.0)	14 (28.0)	14 (28.0)	12 (24.0)	0
		No	1 (2.0)	0	2 (4.0)	7 (14.0)	0	0	15 (30.0)	3 (6.0)	6 (12.0)	6 (12.0)	8 (16.0)	13 (26.0)
	**Combined n (%)**	Yes	73 (80.2)	75 (82.4)	64 (70.3)	41 (45.1)	73 (80.2)	75 (82.4)	40 (44.0)	62 (68.1)	54 (59.3)	53 (58.2)	58 (63.7)	72 (79.1)
		Not sure	14 (15.4)	14 (15.4)	21 (23.1)	30 (33.0)	17 (18.7)	15 (16.5)	25 (27.5)	20 (22.0)	27 (29.7)	28 (30.8)	20 (22.0)	0
		No	4 (4.4)	2 (2.2)	6 (6.6)	20 (22.0)	1 (1.1)	1 (1.1)	26 (28.6)	9 (9.9)	10 (11.0)	10 (11.0)	13 (14.3)	19 (20.9)

Preferences for return of results (incidental findings)

			I want to know about the genetic change if it is medically actionable (can alter my health management or treatment)	I want to know about the genetic change if it is non-medically actionable (will not alter my health management or treatment)	I want to know if I am a carrier of a genetic change that can cause disease (it might affect decisions I make about having children, or for my grandchildren)	I'd like my medical team and the testing laboratory to decide whether I should be told about medically actionable, non-medically actionable and carrier status findings on a case by case basis	I would like a summary of the main findings of my genomic testing report securely stored in CTRL, so I can access it at any time
**Prospective**	**Paediatric n (%)**	Yes	5 (71.4)	4 (57.1)	5 (71.4)	2 (28.6)	5 (71.4)
		Not sure	2 (28.6)	3 (42.9)	2 (28.6)	3 (42.9)	2 (28.6)
		No	0	0	0	2 (28.6)	0
	**Adult n (%)**	Yes	25 (89.3)	20 (71.4)	25 (89.3)	9 (32.1)	26 (92.9)
		Not sure	3 (10.7)	4 (14.3)	2 (7.1)	9 (32.1)	2 (7.1)
		No	0	4 (14.3)	1 (3.6)	10 (35.7)	0
**Retrospective**	**Paediatric n (%)**	Yes	4 (66.7)	4 (66.7)	4 (66.7)	2 (33.3)	5 (83.3)
		Not sure	2 (33.3)	2 (33.3)	2 (33.3)	2 (33.3)	1 (16.7)
		No	0	0	0	2 (33.3)	0
	**Adult n (%)**	Yes	43 (86.0)	34 (68.0)	43 (86.0)	23 (46.0)	43 (86.0)
		Not sure	7 (14.0)	15 (30.0)	6 (12.0)	14 (28.0)	7 (14.0)
		No	0	1 (2.0)	1 (2.0)	13 (26.0)	0
	**Combined n (%)**	Yes	77 (84.6)	62 (68.1)	77 (84.6)	36 (39.6)	79 (86.8)
		Not sure	14 (15.4)	24 (26.4)	12 (13.2)	28 (30.8)	12 (13.2)
		No	0	5 (5.5)	2 (2.2)	27 (29.7)	0

Supplementary Figure 1. CTRL as part of the recruitment strategy

Cardiovascular Genetic Disorders Flagship

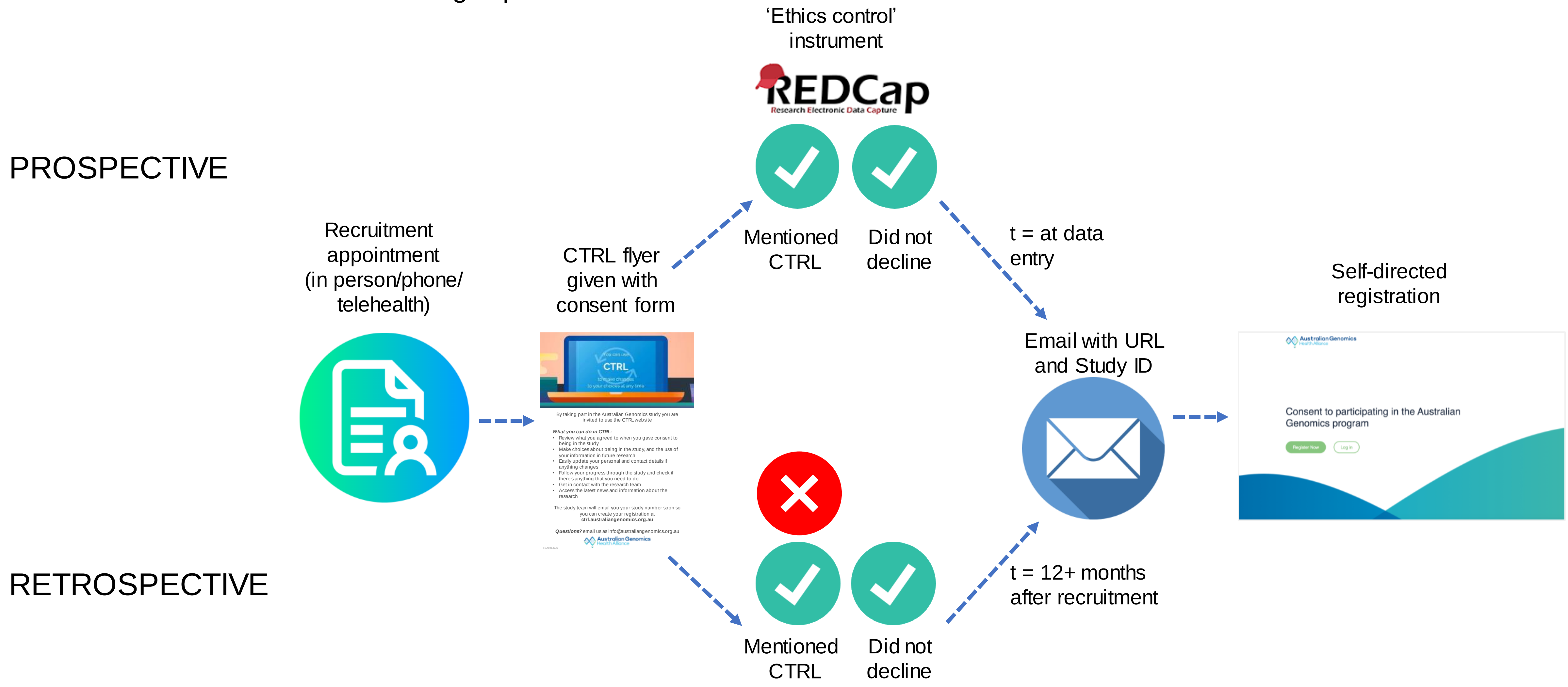

Supplementary Figure 2. Research experience of CTRL and non-CTRL users

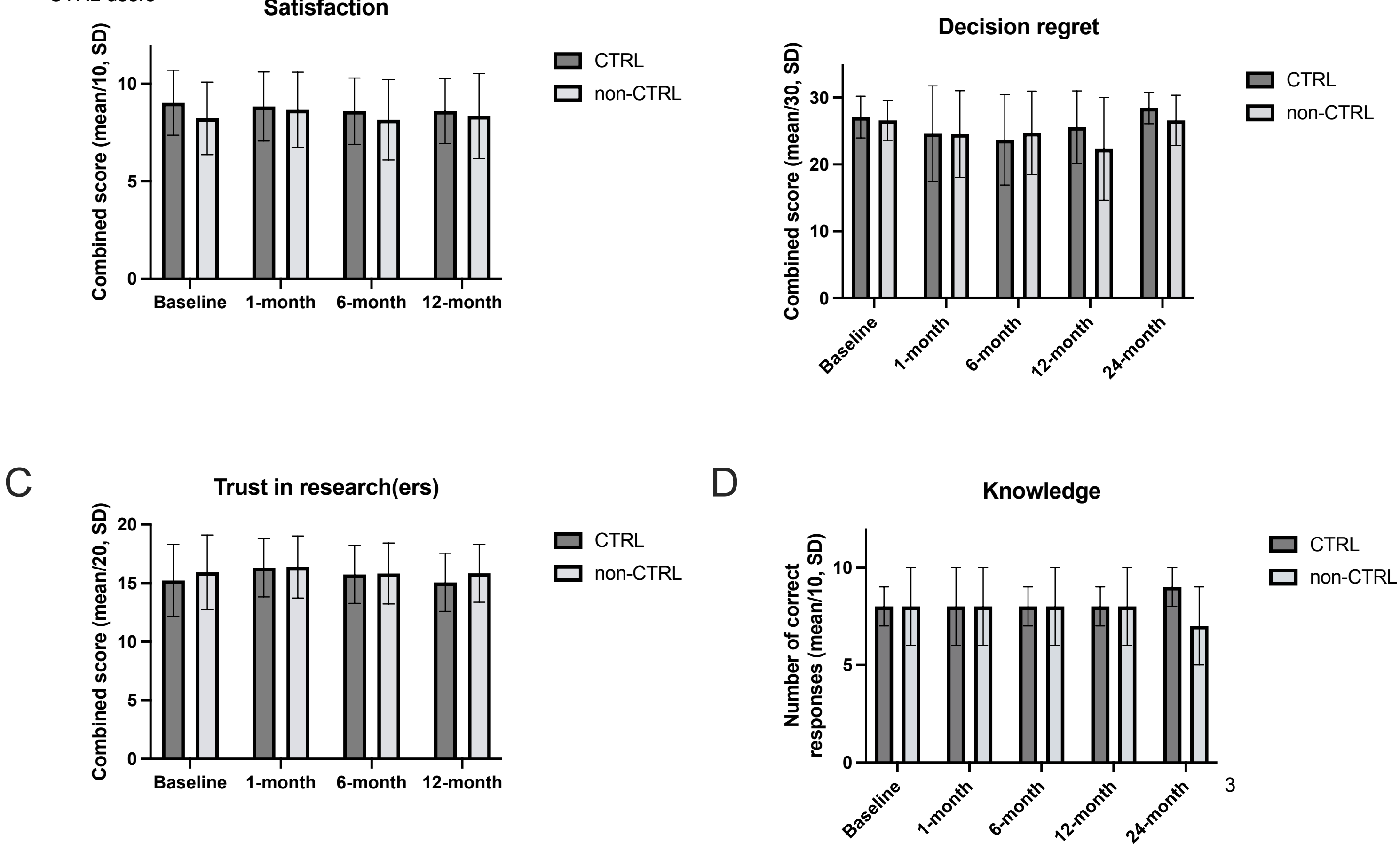

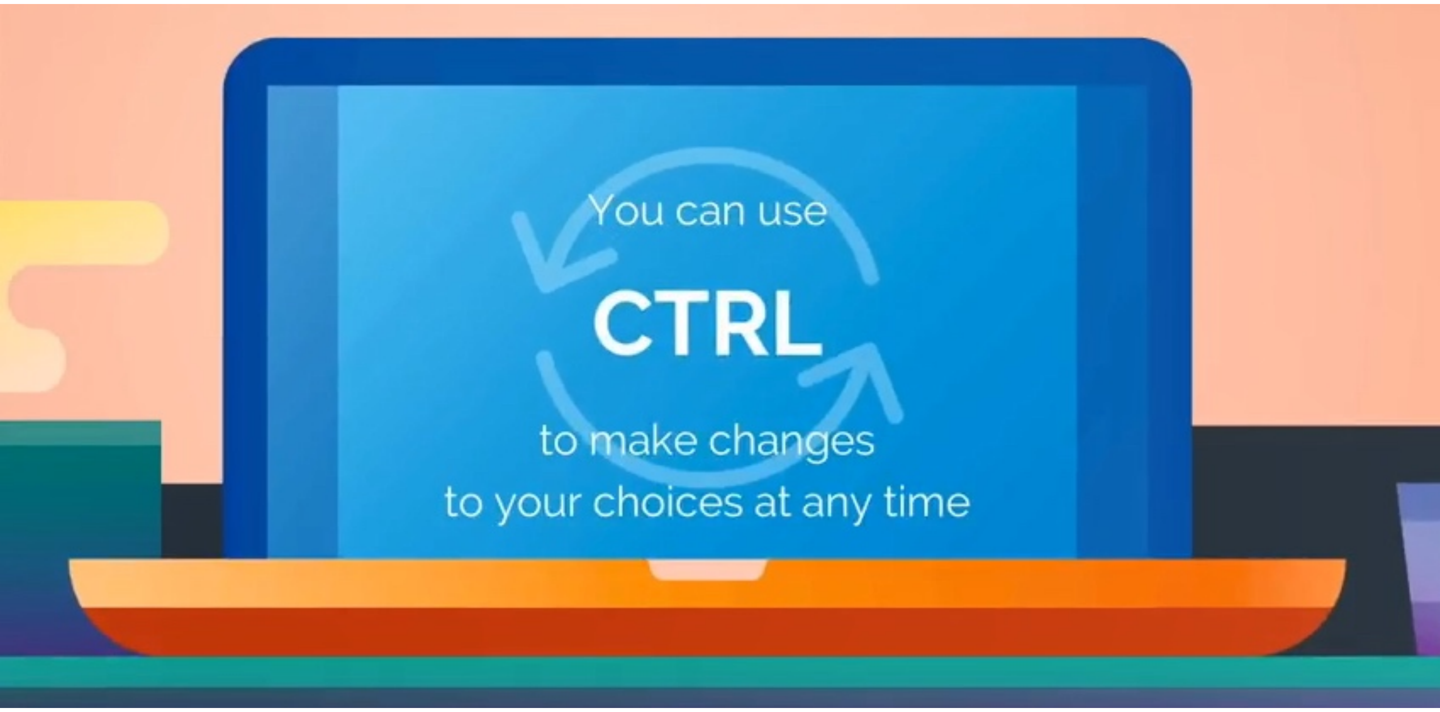

By taking part in the Australian Genomics study you are invited to use the CTRL website

What you can do in CTRL:

- Review what you agreed to when you gave consent to being in the study
- Make choices about being in the study, and the use of your information in future research
- Easily update your personal and contact details if anything changes
- Follow your progress through the study and check if there's anything that you need to do
- Get in contact with the research team
- Access the latest news and information about the research

The study team will email you your study number soon so you can create your registration at **ctrl.australiangenomics.org.au**

Questions? email us as info@australiangenomics.org.au

Australian Genomics Health Alliance

V1 20.02.2020

	Options	Type of choice
**Step 1. Information**		
Watch our short video about the consent process for taking part in medical research, and how you can use this website during the study.		
**Step 2. Consent to the genomic test**		
I understand I am providing a sample so that my DNA can be extracted and all of my genes could be looked at. This is called a genomic test.	Checkbox agreement	Must agree with the statement to participate
I understand have a genomic test may not find the genetic cause of my condition.	Checkbox agreement	Must agree with the statement to participate
I understand my sample and genomic information will be stored by the testing laboratory so that it could be looked at again in the future, when we know more about genes that cause my health condition.	Checkbox agreement	Must agree with the statement to participate
I understand my genomic test results are confidential and will only be shared with the medical team directly involved in my care.	Checkbox agreement	Must agree with the statement to participate
I understand that the testing laboratory could share my anonymous genomic information with other laboratories, in order to understand more about the genetic changes that can cause my health condition or other conditions.	Checkbox agreement	Must agree with the statement to participate
I understand the laboratory performing my test will store and may sometimes use my DNA sample for purposes like references and control material.	Checkbox agreement	Must agree with the statement to participate
I understand there are certain risks involved in having a genomic test. These could include finding out something that may be important for my family's health, incidental findings, or having to disclose the genomic information.	Checkbox agreement	Must agree with the statement to participate
I understand that if I am taking out a new policy for life, critical illness or income protection insurance, the insurer may ask me to disclose that I have had a genetic test and require me to provide my results.	Checkbox agreement	Must agree with the statement to participate
I understand my employer, or future employers, may ask for my results to decide whether there is a risk associated with a particular job role.	Checkbox agreement	Must agree with the statement to participate
I understand that it might be important to tell my family members about genetic changes that could directly affect their health. My medical team will support me in sharing such information with my family.	Checkbox agreement	Must agree with the statement to participate

I understand all of the information above and the choices I have made. (leaving this box unchecked means you will be contacted by a Genetic Counsellor who will give you more information)	Checkbox	May be left unchecked
**Step 3. Consent to research participation**		
I understand that the researchers will collect information from my medical records.	Checkbox agreement	Must agree with the statement to participate
I understand that at times during the research project I will be asked to complete surveys or questionnaires. I'll be reminded when they are ready to complete and I can ask for help to do them.	Checkbox agreement	Must agree with the statement to participate
I understand that my samples and data may be used in further research when no cause is found as part of the initial test and/or when further investigation is required to understand my condition.	Checkbox agreement	Must agree with the statement to participate
I understand all of the information about and the choices I have made. (leaving this box unchecked means you will be contacted by a Genetic Counsellor who will give you more information)	Checkbox	May be left unchecked
**Step 4. Preferences about your results**		
I want to know about the genetic change if it is medically actionable (can alter my health management or treatment).	Yes<br>No<br>Not sure	Optional and can change over time
I want to know about the genetic change if it is non-medically actionable (will not alter my health management or treatment).	Yes<br>No<br>Not sure	Optional and can change over time
I want to know if I am a carrier of a genetic change that can cause disease (it might affect decisions I make about having children, or for my grandchildren).	Yes<br>No<br>Not sure	Optional and can change over time
I'd like my medical team and the testing laboratory to decide whether I should be told about medically actionable, non-medically actionable and carrier status findings on a case by case basis.	Checkbox	Optional and can change over time / may be left unchecked
I would like a summary of the main findings of my genomic testing report securely stored in CTRL, so I can access it at any time.	Checkbox	Optional and can change over time / may be left unchecked

I understand all of the information about and the choices I have made. (leaving this box unchecked means you will be contacted by a Genetic Counsellor who will give you more information)	Checkbox	May be left unchecked
**Step 5. Consent to research outside this study**		
Who can have access to my de-identified samples and information?		
Not-for-profit research organisations (eg Murdoch Children's Research Institute)	Yes<br>No<br>Not sure	Optional and can change over time
Universities and research institutes (eg The University of Queensland)	Yes<br>No<br>Not sure	Optional and can change over time
Government (eg Australian Government Department of Health)	Yes<br>No<br>Not sure	Optional and can change over time
Commercial companies (eg pharmaceutical companies)	Yes<br>No<br>Not sure	Optional and can change over time
What kinds of research can they do with my de-identified samples and information?		
General research use and clinical care	Yes<br>No<br>Not sure	Optional and can change over time
Health/medical/biomedical research	Yes<br>No<br>Not sure	Optional and can change over time
Research must be specifically related to my condition.	Yes<br>No<br>Not sure	Optional and can change over time

Population and ancestry research	Yes<br>No<br>Not sure	Optional and can change over time
I agree to my general health information (eg just my MRIs, blood test or other results) being shared with ither research studies that don't need my genetic information.	Checkbox	Optional and can change over time / may be left unchecked
I agree to my self-reported information (eg questionnaire responses) being shared with other research studies that don't need my genomic information.	Checkbox	Optional and can change over time / may be left unchecked
I want to be contacted every time my de-identified DNA sample, genomic, health or self-reported information is shared.	Checkbox	Optional and can change over time / may be left unchecked
I agree to Australian Genomics sharing my contact details with other research projects and clinical trials doing studies I am eligible for.	Checkbox	Optional and can change over time / may be left unchecked
I understand all of the information above and the choices I have made. (unchecked means you will be contacted by a Genetic Counsellor who will give you more information)	Checkbox	May be left unchecked

Data analysis in Stata IC 17.0

1. **Cohort characteristics (age)**
   a. **Prospective cohort**

```
. summarize age_prospective, detail

                     Age (Prospective)
-------------------------------------------------------------
      Percentiles      Smallest
 1%            0              0
 5%            0              0
10%            7              3       Obs                  35
25%           23              7       Sum of wgt.          35

50%           43                      Mean           37.11429
                        Largest       Std. dev.      19.96581
75%           54             59
90%           59             63       Variance       398.6336
95%           66             66       Skewness      -.4387814
99%           69             69       Kurtosis       2.125167

. tabulate age_prospective_gr

        Age |
  (grouped) |
(Prospectiv |
         e) |      Freq.     Percent        Cum.
------------+-----------------------------------
       0-25 |          9       25.71       25.71
      26-50 |         15       42.86       68.57
        >50 |         11       31.43      100.00
------------+-----------------------------------
      Total |         35      100.00
```

   b. **Retrospective cohort**

```
. summarize age_retrospective, detail

                     Age (Retrospective)
-------------------------------------------------------------
      Percentiles      Smallest
 1%            0              0
 5%            1              0
10%           13              1       Obs                  56
25%           26              3       Sum of wgt.          56

50%           42                      Mean           39.39286
                        Largest       Std. dev.      17.90447
75%           51             63
90%           60             64       Variance       320.5701
95%           64             67       Skewness      -.5787178
99%           72             72       Kurtosis       2.712761

. tabulate age_retrospective_gr

        Age |
  (grouped) |
(Retrospect |
       ive) |      Freq.     Percent        Cum.
------------+-----------------------------------
       0-25 |         14       25.00       25.00
      26-50 |         27       48.21       73.21
        >50 |         15       26.79      100.00
------------+-----------------------------------
      Total |         56      100.00
```

c. Non-CTRL cohort

```
. summarize age_nonCTRL, detail

                       Age (non-CTRL)
-------------------------------------------------------------
      Percentiles      Smallest
 1%            0              0
 5%            0              0
10%            5              0       Obs                 444
25%         24.5              0       Sum of wgt.         444

50%           40                      Mean           37.53153
                        Largest       Std. dev.      19.98795
75%           52             77
90%           63             78       Variance       399.5182
95%           68             81       Skewness      -.2682072
99%           75             82       Kurtosis        2.31126

. tabulate age_nonCTRL_gr

        Age |
  (grouped) |
 (non-CTRL) |      Freq.     Percent        Cum.
------------+-----------------------------------
       0-25 |        115       25.90       25.90
      26-50 |        206       46.40       72.30
        >50 |        123       27.70      100.00
------------+-----------------------------------
      Total |        444      100.00
```

d. Active decliner cohort

```
. summarize age_decl, detail

                          age_decl
-------------------------------------------------------------
      Percentiles      Smallest
 1%            0              0
 5%            0              0
10%            0              0       Obs                  66
25%           22              0       Sum of wgt.          66

50%           38                      Mean           35.18182
                        Largest       Std. dev.      19.46667
75%           49             65
90%           60             68       Variance        378.951
95%           65             72       Skewness       -.317371
99%           72             72       Kurtosis       2.449304

. tabulate age_decl_gr

        Age |
  (grouped) |
(decliners) |      Freq.     Percent        Cum.
------------+-----------------------------------
       0-25 |         17       25.76       25.76
      26-50 |         36       54.55       80.30
        >50 |         13       19.70      100.00
------------+-----------------------------------
      Total |         66      100.00
```

e. Chi-square test for individual ages in the prospective and retrospective cohorts

```
. csgof age_prospective_gr, expperc(26 50 24)
```

age_pr~r	expperc	expfreq	obsfreq
0-25	26	9.1	9
26-50	50	17.5	15
>50	24	8.4	11

chisq(2) is **1.16**, p = **.5591**

```
. csgof age_retrospective_gr, expperc(26 46 28)
```

age_re~r	expperc	expfreq	obsfreq
0-25	26	14.56	14
26-50	46	25.76	27
>50	28	15.68	15

chisq(2) is **.11**, p = **.9461**

f. Chi-square test for individual gender in the prospective and retrospective cohorts

```
. csgof gender_prospective, expperc(46,54)
```

gender~e	expperc	expfreq	obsfreq
Male	46	16.1	17
Female	54	18.9	18

chisq(1) is **.09**, p = **.7602**

```
. csgof gender_retrospective, expperc(55,45)
```

gender~e	expperc	expfreq	obsfreq
Male	55	30.8	39
Female	45	25.2	17

chisq(1) is **4.85**, p = **.0276**

g. Chi-square test for individual education level in the prospective and retrospective cohorts

```
. csgof edu_prospective_gr, expperc(70 30)
```

edu_prospective_gr	expperc	expfreq	obsfreq
Higher education	70	22.4	24
No higher education	30	9.6	8

chisq(1) is **.38**, p = **.5371**

```
. csgof edu_retrospective_gr, expperc(72 28)
```

edu_retrospective~r	expperc	expfreq	obsfreq
Higher education	72	33.84	37
No higher education	28	13.16	10

chisq(1) is **1.05**, p = **.3046**

h. Chi-square test to compare individual education level between those who registered to CTRL and those who didn't

```
. tabulate edu_cohort edu_reg_nonCTRL_gr, chi2
```

	Education (Reg/nonCTRL)		
Cohort	Higher	No higher	Total
Registered	**61**	**18**	**79**
nonCTRL	**200**	**87**	**287**
Total	**261**	**105**	**366**

Pearson chi2(**1**) = **1.7164** Pr = **0.190**

i. Chi-square test on genetic diagnosis in the retrospective cohort

```
. csgof dx_retrospective,expperc redacted
```

dx_retrospective	expperc	expfreq	obsfreq
Postitive diagnosis	redacted	19.04	24
Negative or uncertain diagnosis	redacted	36.96	32

chisq(1) is **1.96**, p = **.1618**

2. Preferences for return of results

a. Prospective adult group

```
. table res_ret_actionable_adult, statistic(frequency) statistic(percent) nformat(%2.1f)
```

	Frequency	Percent
I want to know about the genetic change if it is medically actionable		
Yes	25.0	89.3
Not sure	3.0	10.7
Total	28.0	100.0

```
. table res_ret_nonactionable_adult, statistic(frequency) statistic(percent) nformat(%2.1f)
```

	Frequency	Percent
I want to know about the genetic change if it is non-medically actionable		
No	4.0	14.3
Yes	20.0	71.4
Not sure	4.0	14.3
Total	28.0	100.0

```
. table res_ret_carrier_adult, statistic(frequency) statistic(percent) nformat(%2.1f)
```

	Frequency	Percent
I want to know if I am a carrier of a genetic change that can cause disease		
No	1.0	3.6
Yes	25.0	89.3
Not sure	2.0	7.1
Total	28.0	100.0

```
. table res_ret_team_decision_adult, statistic(frequency) statistic(percent) nformat(%2.1f)
```

	Frequency	Percent
I'd like my medical team and the testing laboratory to decide		
No	10.0	35.7
Yes	9.0	32.1
Not sure	9.0	32.1
Total	28.0	100.0

```
. table res_ret_summary_upload_adult, statistic(frequency) statistic(percent) nformat(%2.1f)
```

	Frequency	Percent
I would like a summary of the main findings securely stored in CTRL		
Yes	26.0	92.9
Not sure	2.0	7.1
Total	28.0	100.0

b. Prospective paediatric (parent/guardian) group

```
. table res_ret_actionable_paed, statistic(frequency) statistic(percent) nformat(%2.1f)
```

	Frequency	Percent
I want to know about the genetic change if it is medically actionable		
Yes	5.0	71.4
Not sure	2.0	28.6
Total	7.0	100.0

```
. table res_ret_nonactionable_paed, statistic(frequency) statistic(percent) nformat(%2.1f)
```

	Frequency	Percent
I want to know about the genetic change if it is non-medically actionable		
Yes	4.0	57.1
Not sure	3.0	42.9
Total	7.0	100.0

```
. table res_ret_carrier_paed, statistic(frequency) statistic(percent) nformat(%2.1f)
```

	Frequency	Percent
I want to know if I am a carrier of a genetic change that can cause disease		
Yes	5.0	71.4
Not sure	2.0	28.6
Total	7.0	100.0

```
. table res_ret_team_decision_paed, statistic(frequency) statistic(percent) nformat(%2.1f)
```

	Frequency	Percent
I'd like my medical team and the testing laboratory to decide		
No	2.0	28.6
Yes	2.0	28.6
Not sure	3.0	42.9
Total	7.0	100.0

```
. table res_ret_summary_upload_paed, statistic(frequency) statistic(percent) nformat(%2.1f)
```

	Frequency	Percent
I would like a summary of the main findings securely stored in CTRL		
Yes	5.0	71.4
Not sure	2.0	28.6
Total	7.0	100.0

c. Retrospective adult group

```
. table res_ret_r_actionable_adult, statistic(frequency) statistic(percent) nformat(%2.1f)
```

	Frequency	Percent
I want to know about the genetic change if it is medically actionable		
Yes	43.0	86.0
Not sure	7.0	14.0
Total	50.0	100.0

```
. table res_ret_r_nonactionable_adult, statistic(frequency) statistic(percent) nformat(%2.1f)
```

	Frequency	Percent
I want to know about the genetic change if it is non-medically actionable		
No	1.0	2.0
Yes	34.0	68.0
Not sure	15.0	30.0
Total	50.0	100.0

```
. table res_ret_r_carrier_adult, statistic(frequency) statistic(percent) nformat(%2.1f)
```

	Frequency	Percent
I want to know if I am a carrier of a genetic change that can cause disease		
No	1.0	2.0
Yes	43.0	86.0
Not sure	6.0	12.0
Total	50.0	100.0

```
. table res_ret_r_team_decision_adult, statistic(frequency) statistic(percent) nformat(%2.1f)
```

	Frequency	Percent
I'd like my medical team and the testing laboratory to decide		
No	13.0	26.0
Yes	23.0	46.0
Not sure	14.0	28.0
Total	50.0	100.0

```
. table res_ret_r_summary_upload_adult, statistic(frequency) statistic(percent) nformat(%2.1f)
```

	Frequency	Percent
I would like a summary of the main findings securely stored in CTRL		
Yes	43.0	86.0
Not sure	7.0	14.0
Total	50.0	100.0

d. Retrospective paediatric (parent/guardian) group

```
. table res_ret_r_actionable_paed, statistic(frequency) statistic(percent) nformat(%2.1f)
```

	Frequency	Percent
I want to know about the genetic change if it is medically actionable		
Yes	4.0	66.7
Not sure	2.0	33.3
Total	6.0	100.0

```
. table res_ret_r_nonactionable_paed, statistic(frequency) statistic(percent) nformat(%2.1f)
```

	Frequency	Percent
I want to know about the genetic change if it is non-medically actionable		
Yes	4.0	66.7
Not sure	2.0	33.3
Total	6.0	100.0

```
. table res_ret_r_carrier_paed, statistic(frequency) statistic(percent) nformat(%2.1f)
```

	Frequency	Percent
I want to know if I am a carrier of a genetic change that can cause disease		
Yes	4.0	66.7
Not sure	2.0	33.3
Total	6.0	100.0

```
. table res_ret_r_team_decision_paed, statistic(frequency) statistic(percent) nformat(%2.1f)
```

	Frequency	Percent
I'd like my medical team and the testing laboratory to decide		
No	2.0	33.3
Yes	2.0	33.3
Not sure	2.0	33.3
Total	6.0	100.0

```
. table res_ret_r_summary_upload_paed, statistic(frequency) statistic(percent) nformat(%2.1f)
```

	Frequency	Percent
I would like a summary of the main findings securely stored in CTRL		
Yes	5.0	83.3
Not sure	1.0	16.7
Total	6.0	100.0

e. Combined (prospective and retrospective)

```
. table res_ret_actionable, statistic(frequency) statistic(percent) nformat(%2.1f)
```

	Frequency	Percent
I want to know about the genetic change if it is medically actionable		
Yes	77.0	84.6
Not sure	14.0	15.4
Total	91.0	100.0

```
. table res_ret_nonactionable, statistic(frequency) statistic(percent) nformat(%2.1f)
```

	Frequency	Percent
I want to know about the genetic change if it is non-medically actionable		
No	5.0	5.5
Yes	62.0	68.1
Not sure	24.0	26.4
Total	91.0	100.0

```
. table res_ret_carrier, statistic(frequency) statistic(percent) nformat(%2.1f)
```

	Frequency	Percent
I want to know if I am a carrier of a genetic change that can cause disease		
No	2.0	2.2
Yes	77.0	84.6
Not sure	12.0	13.2
Total	91.0	100.0

```
. table res_ret_team_decision, statistic(frequency) statistic(percent) nformat(%2.1f)
```

	Frequency	Percent
I'd like my medical team and the testing laboratory to decide		
No	27.0	29.7
Yes	36.0	39.6
Not sure	28.0	30.8
Total	91.0	100.0

```
. table res_ret_summary_upload, statistic(frequency) statistic(percent) nformat(%2.1f)
```

	Frequency	Percent
I would like a summary of the main findings securely stored in CTRL		
Yes	79.0	86.8
Not sure	12.0	13.2
Total	91.0	100.0

3. Preferences for future sample and data use

f. Prospective adult group

```
. table share_nfp_adult, statistic(frequency) statistic(percent) nformat(%2.1f)
```

	Frequency	Percent
Not-for-profit research organisations		
No	2.0	7.1
Yes	23.0	82.1
Not sure	3.0	10.7
Total	28.0	100.0

```
. table share_uni_adult, statistic(frequency) statistic(percent) nformat(%2.1f)
```

	Frequency	Percent
Universities and research institutes		
No	1.0	3.6
Yes	24.0	85.7
Not sure	3.0	10.7
Total	28.0	100.0

```
. table share_gov_adult, statistic(frequency) statistic(percent) nformat(%2.1f)
```

	Frequency	Percent
Government		
No	3.0	10.7
Yes	20.0	71.4
Not sure	5.0	17.9
Total	28.0	100.0

```
. table share_commercial_adult, statistic(frequency) statistic(percent) nformat(%2.1f)
```

	Frequency	Percent
Commercial companies		
No	10.0	35.7
Yes	12.0	42.9
Not sure	6.0	21.4
Total	28.0	100.0

```
. table share_gen_research_adult, statistic(frequency) statistic(percent) nformat(%2.1f)
```

	Frequency	Percent
General research use and clinical care		
Yes	24.0	85.7
Not sure	4.0	14.3
Total	28.0	100.0

```
. table share_hmb_adult, statistic(frequency) statistic(percent) nformat(%2.1f)
```

	Frequency	Percent
Health/medical/biomedical research		
Yes	24.0	85.7
Not sure	4.0	14.3
Total	28.0	100.0

```
. table share_rel_cond_adult, statistic(frequency) statistic(percent) nformat(%2.1f)
```

	Frequency	Percent
Research must be specifically related to my condition		
No	9.0	32.1
Yes	13.0	46.4
Not sure	6.0	21.4
Total	28.0	100.0

```
. table share_pop_anc_adult, statistic(frequency) statistic(percent) nformat(%2.1f)
```

	Frequency	Percent
Population and ancestry research		
No	**5.0**	**17.9**
Yes	**18.0**	**64.3**
Not sure	**5.0**	**17.9**
Total	**28.0**	**100.0**

```
. table share_gen_health_info_adult, statistic(frequency) statistic(percent) nformat(%2.1f)
```

	Frequency	Percent
Agree to my general health information being shared with other research studies		
No	**3.0**	**10.7**
Yes	**17.0**	**60.7**
Not sure	**8.0**	**28.6**
Total	**28.0**	**100.0**

```
. table share_self_rep_info_adult, statistic(frequency) statistic(percent) nformat(%2.1f)
```

	Frequency	Percent
Agree to my self-reported information being shared with other research studies		
No	**3.0**	**10.7**
Yes	**16.0**	**57.1**
Not sure	**9.0**	**32.1**
Total	**28.0**	**100.0**

```
. table share_want_cont_adult, statistic(frequency) statistic(percent) nformat(%2.1f)
```

	Frequency	Percent
I want to be contacted every time my information is shared		
No	**5.0**	**17.9**
Yes	**18.0**	**64.3**
Not sure	**5.0**	**17.9**
Total	**28.0**	**100.0**

```
. table share_cont_details_adult, statistic(frequency) statistic(percent) nformat(%2.1f)
```

	Frequency	Percent
I agree to Aus Genomics sharing my contact details with other research projects		
No	**5.0**	**17.9**
Yes	**23.0**	**82.1**
Total	**28.0**	**100.0**

g. Prospective paediatric (parent/guardian) group

```
. table share_nfp_paed, statistic(frequency) statistic(percent) nformat(%2.1f)
```

	Frequency	Percent
Not-for-profit research organisations		
No	1.0	14.3
Yes	5.0	71.4
Not sure	1.0	14.3
Total	7.0	100.0

```
. table share_uni_paed, statistic(frequency) statistic(percent) nformat(%2.1f)
```

	Frequency	Percent
Universities and research institutes		
No	1.0	14.3
Yes	5.0	71.4
Not sure	1.0	14.3
Total	7.0	100.0

```
. table share_gov_paed, statistic(frequency) statistic(percent) nformat(%2.1f)
```

	Frequency	Percent
Government		
No	1.0	14.3
Yes	4.0	57.1
Not sure	2.0	28.6
Total	7.0	100.0

```
. table share_commercial_paed, statistic(frequency) statistic(percent) nformat(%2.1f)
```

	Frequency	Percent
Commercial companies		
No	2.0	28.6
Yes	3.0	42.9
Not sure	2.0	28.6
Total	7.0	100.0

```
. table share_gen_research_paed, statistic(frequency) statistic(percent) nformat(%2.1f)
```

	Frequency	Percent
General research use and clinical care		
No	1.0	14.3
Yes	4.0	57.1
Not sure	2.0	28.6
Total	7.0	100.0

```
. table share_hmb_paed, statistic(frequency) statistic(percent) nformat(%2.1f)
```

	Frequency	Percent
Health/medical/biomedical research		
No	1.0	14.3
Yes	5.0	71.4
Not sure	1.0	14.3
Total	7.0	100.0

```
. table share_rel_cond_paed, statistic(frequency) statistic(percent) nformat(%2.1f)
```

	Frequency	Percent
Research must be specifically related to my condition		
No	1.0	14.3
Yes	4.0	57.1
Not sure	2.0	28.6
Total	7.0	100.0

```
. table share_pop_anc_paed, statistic(frequency) statistic(percent) nformat(%2.1f)
```

	Frequency	Percent
Population and ancestry research		
No	**1.0**	**14.3**
Yes	**3.0**	**42.9**
Not sure	**3.0**	**42.9**
Total	**7.0**	**100.0**

```
. table share_gen_health_info_paed, statistic(frequency) statistic(percent) nformat(%2.1f)
```

	Frequency	Percent
Agree to my general health information being shared with other research studies		
No	**1.0**	**14.3**
Yes	**4.0**	**57.1**
Not sure	**2.0**	**28.6**
Total	**7.0**	**100.0**

```
. table share_self_rep_info_paed, statistic(frequency) statistic(percent) nformat(%2.1f)
```

	Frequency	Percent
Agree to my self-reported information being shared with other research studies		
No	**1.0**	**14.3**
Yes	**4.0**	**57.1**
Not sure	**2.0**	**28.6**
Total	**7.0**	**100.0**

```
. table share_want_cont_paed, statistic(frequency) statistic(percent) nformat(%2.1f)
```

	Frequency	Percent
I want to be contacted every time my information is shared		
Yes	**6.0**	**85.7**
Not sure	**1.0**	**14.3**
Total	**7.0**	**100.0**

```
. table share_cont_details_paed, statistic(frequency) statistic(percent) nformat(%2.1f)
```

	Frequency	Percent
I agree to Aus Genomics sharing my contact details with other research projects		
Yes	**7.0**	**100.0**
Total	**7.0**	**100.0**

h. Retrospective adult group

```
. table share_r_nfp_adult, statistic(frequency) statistic(percent) nformat(%2.1f)
```

	Frequency	Percent
Not-for-profit research organisations		
No	1.0	2.0
Yes	40.0	80.0
Not sure	9.0	18.0
Total	50.0	100.0

```
. table share_r_uni_adult, statistic(frequency) statistic(percent) nformat(%2.1f)
```

	Frequency	Percent
Universities and research institutes		
Yes	41.0	82.0
Not sure	9.0	18.0
Total	50.0	100.0

```
. table share_r_gov_adult, statistic(frequency) statistic(percent) nformat(%2.1f)
```

	Frequency	Percent
Government		
No	2.0	4.0
Yes	36.0	72.0
Not sure	12.0	24.0
Total	50.0	100.0

```
. table share_r_commercial_adult, statistic(frequency) statistic(percent) nformat(%2.1f)
```

	Frequency	Percent
Commercial companies		
No	7.0	14.0
Yes	24.0	48.0
Not sure	19.0	38.0
Total	50.0	100.0

```
. table share_r_gen_research_adult, statistic(frequency) statistic(percent) nformat(%2.1f)
```

	Frequency	Percent
General research use and clinical care		
Yes	40.0	80.0
Not sure	10.0	20.0
Total	50.0	100.0

```
. table share_r_hmb_adult, statistic(frequency) statistic(percent) nformat(%2.1f)
```

	Frequency	Percent
Health/medical/biomedical research		
Yes	41.0	82.0
Not sure	9.0	18.0
Total	50.0	100.0

```
. table share_r_rel_cond_adult, statistic(frequency) statistic(percent) nformat(%2.1f)
```

	Frequency	Percent
Research must be specifically related to my condition		
No	15.0	30.0
Yes	20.0	40.0
Not sure	15.0	30.0
Total	50.0	100.0

```
. table share_r_pop_anc_adult, statistic(frequency) statistic(percent) nformat(%2.1f)
```

	Frequency	Percent
Population and ancestry research		
No	**3.0**	**6.0**
Yes	**36.0**	**72.0**
Not sure	**11.0**	**22.0**
Total	**50.0**	**100.0**

```
. table share_r_gen_health_info_adult, statistic(frequency) statistic(percent) nformat(%2.1f)
```

	Frequency	Percent
Agree to my general health information being shared with other research studies		
No	**6.0**	**12.0**
Yes	**30.0**	**60.0**
Not sure	**14.0**	**28.0**
Total	**50.0**	**100.0**

```
. table share_r_self_rep_info_adult, statistic(frequency) statistic(percent) nformat(%2.1f)
```

	Frequency	Percent
Agree to my self-reported information being shared with other research studies		
No	**6.0**	**12.0**
Yes	**30.0**	**60.0**
Not sure	**14.0**	**28.0**
Total	**50.0**	**100.0**

```
. table share_r_want_cont_adult, statistic(frequency) statistic(percent) nformat(%2.1f)
```

	Frequency	Percent
I want to be contacted every time my information is shared		
No	**8.0**	**16.0**
Yes	**30.0**	**60.0**
Not sure	**12.0**	**24.0**
Total	**50.0**	**100.0**

```
. table share_r_cont_details_adult, statistic(frequency) statistic(percent) nformat(%2.1f)
```

	Frequency	Percent
I agree to Aus Genomics sharing my contact details with other research projects		
No	**13.0**	**26.0**
Yes	**37.0**	**74.0**
Total	**50.0**	**100.0**

i. Retrospective paediatric (parent/guardian) group

```
. table share_r_nfp_paed, statistic(frequency) statistic(percent) nformat(%2.1f)
```

	Frequency	Percent
Not-for-profit research organisations		
Yes	5.0	83.3
Not sure	1.0	16.7
Total	6.0	100.0

```
. table share_r_uni_paed, statistic(frequency) statistic(percent) nformat(%2.1f)
```

	Frequency	Percent
Universities and research institutes		
Yes	5.0	83.3
Not sure	1.0	16.7
Total	6.0	100.0

```
. table share_r_gov_paed, statistic(frequency) statistic(percent) nformat(%2.1f)
```

	Frequency	Percent
Government		
Yes	4.0	66.7
Not sure	2.0	33.3
Total	6.0	100.0

```
. table share_r_commercial_paed, statistic(frequency) statistic(percent) nformat(%2.1f)
```

	Frequency	Percent
Commercial companies		
No	1.0	16.7
Yes	2.0	33.3
Not sure	3.0	50.0
Total	6.0	100.0

```
. table share_r_gen_research_paed, statistic(frequency) statistic(percent) nformat(%2.1f)
```

	Frequency	Percent
General research use and clinical care		
Yes	5.0	83.3
Not sure	1.0	16.7
Total	6.0	100.0

```
. table share_r_hmb_paed, statistic(frequency) statistic(percent) nformat(%2.1f)
```

	Frequency	Percent
Health/medical/biomedical research		
Yes	5.0	83.3
Not sure	1.0	16.7
Total	6.0	100.0

```
. table share_r_rel_cond_paed, statistic(frequency) statistic(percent) nformat(%2.1f)
```

	Frequency	Percent
Research must be specifically related to my condition		
No	1.0	16.7
Yes	3.0	50.0
Not sure	2.0	33.3
Total	6.0	100.0

```
. table share_r_pop_anc_paed, statistic(frequency) statistic(percent) nformat(%2.1f)
```

	Frequency	Percent
Population and ancestry research		
Yes	5.0	83.3
Not sure	1.0	16.7
Total	6.0	100.0

```
. table share_r_gen_health_info_paed, statistic(frequency) statistic(percent) nformat(%2.1f)
```

	Frequency	Percent
Agree to my general health information being shared with other research studies		
Yes	3.0	50.0
Not sure	3.0	50.0
Total	6.0	100.0

```
. table share_r_self_rep_info_paed, statistic(frequency) statistic(percent) nformat(%2.1f)
```

	Frequency	Percent
Agree to my self-reported information being shared with other research studies		
Yes	3.0	50.0
Not sure	3.0	50.0
Total	6.0	100.0

```
. table share_r_want_cont_paed, statistic(frequency) statistic(percent) nformat(%2.1f)
```

	Frequency	Percent
I want to be contacted every time my information is shared		
Yes	4.0	66.7
Not sure	2.0	33.3
Total	6.0	100.0

```
. table share_r_cont_details_paed, statistic(frequency) statistic(percent) nformat(%2.1f)
```

	Frequency	Percent
I agree to Aus Genomics sharing my contact details with other research projects		
No	1.0	16.7
Yes	5.0	83.3
Total	6.0	100.0

j. Combined (prospective and retrospective)

```
. table share_nfp, statistic(frequency) statistic(percent) nformat(%2.1f)
```

	Frequency	Percent
Not-for-profit research organisations		
No	4.0	4.4
Yes	73.0	80.2
Not sure	14.0	15.4
Total	91.0	100.0

```
. table share_uni, statistic(frequency) statistic(percent) nformat(%2.1f)
```

	Frequency	Percent
Universities and research institutes		
No	2.0	2.2
Yes	75.0	82.4
Not sure	14.0	15.4
Total	91.0	100.0

```
. table share_gov, statistic(frequency) statistic(percent) nformat(%2.1f)
```

	Frequency	Percent
Government		
No	6.0	6.6
Yes	64.0	70.3
Not sure	21.0	23.1
Total	91.0	100.0

```
. table share_commercial, statistic(frequency) statistic(percent) nformat(%2.1f)
```

	Frequency	Percent
Commercial companies		
No	20.0	22.0
Yes	41.0	45.1
Not sure	30.0	33.0
Total	91.0	100.0

```
. table share_gen_research, statistic(frequency) statistic(percent) nformat(%2.1f)
```

	Frequency	Percent
General research use and clinical care		
No	1.0	1.1
Yes	73.0	80.2
Not sure	17.0	18.7
Total	91.0	100.0

```
. table share_hmb, statistic(frequency) statistic(percent) nformat(%2.1f)
```

	Frequency	Percent
Health/medical/biomedical research		
No	1.0	1.1
Yes	75.0	82.4
Not sure	15.0	16.5
Total	91.0	100.0

```
. table share_rel_cond, statistic(frequency) statistic(percent) nformat(%2.1f)
```

	Frequency	Percent
Research must be specifically related to my condition		
No	26.0	28.6
Yes	40.0	44.0
Not sure	25.0	27.5
Total	91.0	100.0

```
. table share_pop_anc, statistic(frequency) statistic(percent) nformat(%2.1f)
```

	Frequency	Percent
Population and ancestry research		
No	9.0	9.9
Yes	62.0	68.1
Not sure	20.0	22.0
Total	91.0	100.0

```
. table share_gen_health_info, statistic(frequency) statistic(percent) nformat(%2.1f)
```

	Frequency	Percent
Agree to my general health information being shared with other research studies		
No	10.0	11.0
Yes	54.0	59.3
Not sure	27.0	29.7
Total	91.0	100.0

```
. table share_self_rep_info, statistic(frequency) statistic(percent) nformat(%2.1f)
```

	Frequency	Percent
Agree to my self-reported information being shared with other research studies		
No	10.0	11.0
Yes	53.0	58.2
Not sure	28.0	30.8
Total	91.0	100.0

```
. table share_want_cont, statistic(frequency) statistic(percent) nformat(%2.1f)
```

	Frequency	Percent
I want to be contacted every time my information is shared		
No	13.0	14.3
Yes	58.0	63.7
Not sure	20.0	22.0
Total	91.0	100.0

```
. table share_cont_details, statistic(frequency) statistic(percent) nformat(%2.1f)
```

	Frequency	Percent
I agree to Aus Genomics sharing my contact details with other research projects		
No	19.0	20.9
Yes	72.0	79.1
Total	91.0	100.0

4. Comparison of preferences between the prospective and the retrospective cohorts

```
. tabulate res_ret_actionable cohort, chi2

  I want to
 know about
        the
    genetic
  change if
      it is
  medically         Cohort
 actionable   Prospecti  Retrospec       Total

        Yes          30         47          77
   Not sure           5          9          14

      Total          35         56          91

          Pearson chi2(1) =   0.0528   Pr = 0.818

. tabulate res_ret_nonactionable cohort, chi2

  I want to
 know about
        the
    genetic
  change if
      it is
non-medica
       lly          Cohort
 actionable   Prospecti  Retrospec       Total

         No           4          1           5
        Yes          24         38          62
   Not sure           7         17          24

      Total          35         56          91

          Pearson chi2(2) =   4.5227   Pr = 0.104

. tabulate res_ret_carrier cohort, chi2

  I want to
  know if I
       am a
 carrier of
  a genetic
     change
   that can
      cause         Cohort
    disease   Prospecti  Retrospec       Total

         No           1          1           2
        Yes          30         47          77
   Not sure           4          8          12

      Total          35         56          91

          Pearson chi2(2) =   0.2540   Pr = 0.881
```

```
. tabulate res_ret_team_decision cohort, chi2
```

I'd like my medical team and the testing laboratory to decide	Cohort Prospecti	Retrospec	Total
No	12	15	27
Yes	11	25	36
Not sure	12	16	28
Total	35	56	91

Pearson chi2(2) = 1.5876 Pr = 0.452

```
. tabulate res_ret_summary_upload cohort, chi2
```

I would like a summary of the main findings securely stored in CTRL	Cohort Prospecti	Retrospec	Total
Yes	31	48	79
Not sure	4	8	12
Total	35	56	91

Pearson chi2(1) = 0.1536 Pr = 0.695

```
. tabulate share_nfp cohort, chi2
```

Not-for-profit research organisations	Cohort Prospecti	Retrospec	Total
No	3	1	4
Yes	28	45	73
Not sure	4	10	14
Total	35	56	91

Pearson chi2(2) = 2.8352 Pr = 0.242

```
. tabulate share_uni cohort, chi2
```

Universities and research institutes	Cohort Prospecti	Retrospec	Total
No	2	0	2
Yes	29	46	75
Not sure	4	10	14
Total	35	56	91

Pearson chi2(2) = 3.7799 Pr = 0.151

```
. tabulate share_gov cohort, chi2
```

Government	Cohort Prospecti	Retrospec	Total
No	4	2	6
Yes	24	40	64
Not sure	7	14	21
Total	35	56	91

Pearson chi2(2) = 2.2750 Pr = 0.321

```
. tabulate share_commercial cohort, chi2
```

Commercial companies	Cohort Prospecti	Retrospec	Total
No	12	8	20
Yes	15	26	41
Not sure	8	22	30
Total	35	56	91

Pearson chi2(2) = 5.7443 Pr = 0.057

```
. tabulate share_gen_research cohort, chi2

    General |
   research |
    use and |
   clinical |        Cohort
       care | Prospecti  Retrospec |     Total
------------+----------------------+----------
         No |         1          0 |         1 
        Yes |        28         45 |        73 
   Not sure |         6         11 |        17 
------------+----------------------+----------
      Total |        35         56 |        91 

          Pearson chi2(2) =   1.6724   Pr = 0.433

. tabulate share_hmb cohort, chi2

Health/med |
ical/biome |
      dical |        Cohort
   research | Prospecti  Retrospec |     Total
------------+----------------------+----------
         No |         1          0 |         1 
        Yes |        29         46 |        75 
   Not sure |         5         10 |        15 
------------+----------------------+----------
      Total |        35         56 |        91 

          Pearson chi2(2) =   1.7680   Pr = 0.413

. tabulate share_rel_cond cohort, chi2

   Research |
    must be |
specifical |
ly related |
      to my |        Cohort
  condition | Prospecti  Retrospec |     Total
------------+----------------------+----------
         No |        10         16 |        26 
        Yes |        17         23 |        40 
   Not sure |         8         17 |        25 
------------+----------------------+----------
      Total |        35         56 |        91 

          Pearson chi2(2) =   0.7166   Pr = 0.699
```

```
. tabulate share_pop_anc cohort, chi2
```

Population and ancestry research	Cohort Prospecti	Retrospec	Total
No	6	3	9
Yes	21	41	62
Not sure	8	12	20
Total	35	56	91

Pearson chi2(2) = 3.5970 Pr = 0.166

```
. tabulate share_gen_health_info cohort, chi2
```

Agree to my general health informatio n being shared with other studies	Cohort Prospecti	Retrospec	Total
No	4	6	10
Yes	21	33	54
Not sure	10	17	27
Total	35	56	91

Pearson chi2(2) = 0.0373 Pr = 0.982

```
. tabulate share_self_rep_info cohort, chi2
```

Agree to my self-repor ted informatio n being shared with other studies	Cohort Prospecti	Retrospec	Total
No	4	6	10
Yes	20	33	53
Not sure	11	17	28
Total	35	56	91

Pearson chi2(2) = 0.0298 Pr = 0.985

. tabulate share_want_cont cohort, chi2

I want to be contacted every time my information is shared	Cohort Prospecti	Retrospec	Total
No	5	8	13
Yes	24	34	58
Not sure	6	14	20
Total	35	56	91

Pearson chi2(2) = 0.8136 Pr = 0.666

. tabulate share_cont_details cohort, chi2

I agree to Aus Genomics sharing my contact details with other projects	Cohort Prospecti	Retrospec	Total
No	5	14	19
Yes	30	42	72
Total	35	56	91

Pearson chi2(1) = 1.4967 Pr = 0.221

5. Self-reported experience evaluation measures

5.1. Satisfaction

a. CTRL group

Variable	Obs	Mean	Std. Dev.	Min	Max
satisfacti~l	39	9.025641	1.662072	3	10
satisfact~1m	36	8.833333	1.764734	2	10
satisfact~6m	20	8.6	1.698296	5	10
satisfac~12m	20	8.6	1.667018	5	10

b. Non-CTRL group

Variable	Obs	Mean	Std. Dev.	Min	Max
satisfacti~l	161	8.223602	1.857334	2	10
satisfact~1m	123	8.658537	1.928345	0	10
satisfact~6m	68	8.147059	2.060754	1	10
satisfac~12m	44	8.340909	2.177509	1	10

5.2. Decision regret

a. CTRL group

BASELINE

Variable	Obs	Mean	Std. Dev.	Min	Max
decision_c~l	40	27.075	3.116437	20	30

1-MONTH AFTER RETURN OF RESULT

Variable	Obs	Mean	Std. Dev.	Min	Max
decision_~1m	34	24.58824	7.148289	6	30

6-MONTH AFTER RETURN OF RESULT

Variable	Obs	Mean	Std. Dev.	Min	Max
decision_~6m	19	23.68421	6.758144	6	30

12-MONTH AFTER RETURN OF RESULT

Variable	Obs	Mean	Std. Dev.	Min	Max
decision_~2m	19	25.57895	5.409003	10	30

24-MONTH AFTER RETURN OF RESULT

Variable	Obs	Mean	Std. Dev.	Min	Max
decision_~4m	16	28.4375	2.337199	24	30

b. Non-CTRL group

BASELINE

Variable	Obs	Mean	Std. Dev.	Min	Max
decision_n~l	159	26.59119	2.987768	8	30

1-MONTH AFTER RETURN OF RESULT

Variable	Obs	Mean	Std. Dev.	Min	Max
decision_~1m	119	24.54622	6.469941	6	30

6-MONTH AFTER RETURN OF RESULT

Variable	Obs	Mean	Std. Dev.	Min	Max
decision_~6m	67	24.71642	6.229952	6	30

12-MONTH AFTER RETURN OF RESULT

Variable	Obs	Mean	Std. Dev.	Min	Max
decision_~2m	44	22.34091	7.673401	6	30

24-MONTHS AFTER RETURN OF RESULT

Variable	Obs	Mean	Std. Dev.	Min	Max
decision_~4m	29	26.58621	3.746591	18	30

5.3. Trust in research(ers)

a. CTRL group

BASELINE

Variable	Obs	Mean	Std. Dev.	Min	Max
trust_ctrl~l	39	15.23077	3.064753	4	20

1-MONTH AFTER RETURN OF RESULT

Variable	Obs	Mean	Std. Dev.	Min	Max
trust_ctr~1m	36	16.30556	2.493643	12	20

6-MONTH AFTER RETURN OF RESULT

Variable	Obs	Mean	Std. Dev.	Min	Max
trust_ctr~6m	19	15.73684	2.445906	12	20

12-MONTH AFTER RETURN OF RESULT

Variable	Obs	Mean	Std. Dev.	Min	Max
trust_ct~12m	20	15.05	2.459675	10	20

b. Non-CTRL group

BASELINE

Variable	Obs	Mean	Std. Dev.	Min	Max
trust_nonc~l	158	15.92405	3.181444	4	20

1-MONTH AFTER RETURN OF RESULT

Variable	Obs	Mean	Std. Dev.	Min	Max
trust_non~1m	121	16.38017	2.646558	11	20

6-MONTH AFTER RETURN OF RESULT

Variable	Obs	Mean	Std. Dev.	Min	Max
trust_non~6m	68	15.82353	2.59702	8	20

12-MONTH AFTER RETURN OF RESULT

Variable	Obs	Mean	Std. Dev.	Min	Max
trust_no~12m	44	15.84091	2.458428	11	20

5.4. Understanding of genomic testing

a. CTRL group

Variable	N	Mean	SD	Min	Max
knowledge_sum_ctrl_adult_bl	63	8	1	5	10
knowledge_sum_ctrl_adult_1m	45	8	2	4	10
knowledge_sum_ctrl_adult_6m	36	8	1	4	10
knowledge_sum_ctrl_adult_12m	33	8	1	6	10
knowledge_sum_ctrl_adult_24m	17	9	1	7	10

b. Non-CTRL group

Variable	N	Mean	SD	Min	Max
knowledge_sum_nonCTRL_adult_bl	256	8	2	0	10
knowledge_sum_nonCTRL_adult_1m	152	8	2	2	10
knowledge_sum_nonCTRL_adult_6m	121	8	2	0	10
knowledge_sum_nonCTRL_adult_12m	75	8	2	3	10
knowledge_sum_nonCTRL_adult_24m	28	7	2	0	10

Self-reported experience evaluation measures

A. Satisfaction

Thinking about your involvement in the study so far, overall…

I had a very poor experience										I had a very good experience
0	1	2	3	4	5	6	7	8	9	10

Graham C, MacCormick S. Overarching Questions for Patient Surveys: Development Report for the Care Quality Commission (CQC). Picker Institute Europe. 2012

B. My decision

Please think about the decision you made to participate in the study after talking to the clinician/genetic counsellor.

	Strongly Agree	Agree	Neither Agree Nor Disagree	Disagree	Strongly Disagree
It was the right decision					
I regret the choice that was made					
I would go for the same choice if I had to do it over again					
The choice did me a lot of harm					
The decision was a wise one					
I know I can easily change my mind					

Brehaut JC, O'Connor AM, Wood TJ, et al. Validation of a decision regret scale. *Med Decis Making*. 2003;23(4):281-292. doi:10.1177/0272989X03256005

C. Trust

	Strongly Agree	Agree	Neither Agree Nor Disagree	Disagree	Strongly Disagree
Doctors who do medical research care only about what is best for each participant.					

Doctors tell participants everything they need to know about being in a research study.					
Medical researchers treat people like "guinea pigs."					
I completely trust doctors who do medical research.					

Hall MA, Camacho F, Lawlor JS, Depuy V, Sugarman J, Weinfurt K. Measuring trust in medical researchers. Med Care. 2006;44(11):1048-1053. doi:10.1097/01.mlr.0000228023.37087.cb

D. Your Understanding of Genomic Testing

The following statements relate to your understanding of the genomic sequencing test. Your responses will help us improve the information we provide about genomic sequencing in the future.

	True	False	Unsure
1. The test may identify a gene variant that causes the condition			
2. The test may not identify any gene variants that the doctors think could cause the condition.			
3. The test may identify gene variants that might be the cause of the condition, but the doctors are uncertain.			
4. The test may reveal gene variants that can cause other unrelated conditions that may develop in the future.			
5. The test may find gene variants that could be passed on in the family.			
6. The data from the test cannot be stored and looked at in the future - a new test is required each time.			
7. Anonymised data from the test can be used for the purpose of advancing knowledge (research).			
8. The test will rule out a genetic condition in me.			
9. The main reason for the test is to help my family.			
10. If the test is negative, my children are not at increased risk.			
